# Applying the Poincaré Disc Method to Analyze the Hierarchical Structure of TADs in Hi-C Data

**DOI:** 10.3390/genes17080932

**Published:** 2026-08-10

**Authors:** Andrey Timofeev, Alexander Bratchikov, Alexander Anufriev

**Affiliations:** LLC “AI Center for SCO+ Countries”, St. Petersburg 197341, Russia; bav@centeraisco.com (A.B.); aas@centeraisco.com (A.A.)

**Keywords:** Hi-C data analysis, topological associated domains (TADs), Poincaré disc embedding, hyperbolic geometry, hierarchical chromatin organization, Insulation Score (IS), Directionality Index (DI), isogenic cell pair MCF10A/MCF7, chromatin reorganization in cancer

## Abstract

**Background/Objectives:** The hierarchical organization of topological associating domains (TADs) is a fundamental feature of three-dimensional genome architecture, yet its systematic characterization remains challenging due to the scale-dependent nature of conventional detection methods. Here, we applied hyperbolic embedding into a Poincaré disc to analyze TAD hierarchies from Hi-C data. **Methods:** Genomic loci were projected onto the disc such that the radial coordinate encodes hierarchical depth: subTADs localize near the center, while metaTADs shift toward the periphery. The method was validated on ten cell lines, including the isogenic MCF10A/MCF7 pair, and does not require manual parameter tuning across resolutions. **Results:** In the MCF10A/MCF7 isogenic system, neoplastic transformation was associated with a redistribution of the hierarchy: the number of small TADs (levels 1–3) decreased by 6.7%, whereas large TADs (levels 4–6) increased by 4.7%, with the largest domains (level 6) showing a 33.9% increase. Comparison with standard approaches—Insulation Score (IS) and Directionality Index (DI)—yielded an average Jaccard index of 0.568 and F1 score of 0.718 against DI. **Conclusions:** Unlike IS and DI, which operate at fixed scales, the proposed approach recovers the full TAD hierarchy from a single embedding, enabling cross-resolution and cross-cell-line comparisons without parameter reoptimization. These results demonstrate that the Poincaré disc method provides a robust, interpretable, and scale-invariant framework for detecting hierarchical chromatin rearrangements associated with cancer.

## 1. Introduction

Topological association domains (TADs) are the fundamental units of the three-dimensional organization of the genome. They have a hierarchical structure: subTADs are nested in TADs, which in turn are combined into metaTADs. Existing methods, such as the Insulation Score (IS) [1] and Directionality Index (DI) [2], operate at fixed scales and require manual parameter selection. They effectively detect boundaries at their respective scales, but do not provide a unified view of the hierarchy. The aim of this study is to apply and validate the hyperbolic embedding method, which automatically extracts the TAD hierarchy from a single embedding into a Poincaré disc, where the radial position of the point encodes the depth of the hierarchy. Hyperbolic geometry is naturally suited to tree structures due to the exponential growth of volume with the radius [3]. The novelty and advantages of the proposed approach include:

- Single hierarchical frame—Unlike IS and DI, the method does not require the choice of scale; all levels of the hierarchy are derived from a single embedding.
- Automatic adaptation—The method works at different resolutions without reconfiguring parameters.
- Visualization of a 3D structure in 2D—The Poincaré disc provides an intuitive representation of the organization of the chromatin hierarchy.

A recent paper [4] also used hyperbolic embedding in a Poincaré disc to analyze chromatin contact networks (CCNs) built from Hi-C data. However, the approach of those authors is fundamentally different from that set forth in this work. In [4], a contact graph is constructed based on the correlations of interaction profiles, and the structure of this graph is embedded in the Poincaré disc rather than the Hi-C distance matrix itself. The radial position of the graph nodes is interpreted as the depth of the hierarchy of information flows in a given network model. In contrast to the proposed approach, [4] does not assign discrete hierarchy levels and does not perform clustering for TAD discovery. Furthermore, cancer is considered as only one of several possible processes (along with embryogenesis and differentiation) accompanied by a flattening of the hierarchy. Thus, despite a common mathematical framework, [4] investigates changes in the information architecture of the chromatin network across a wide range of cellular states, while the present paper analyzes the redistribution of TADs at different scales during cancer transformation. These are fundamentally different tasks.

## 2. Materials and Methods

### 2.1. Data

Ten cell lines from three independent sources were analyzed.

The GSE63525 set (8 lines, 100 kb resolution) is shown in Table 1.

**Table 1:** GSE63525 set (8 lines, 100 kb).

Cell Line	Status	Tissue Type
4DNFIZGCA8AH (GM12878)	Healthy	B-lymphocytes (blood)
GM12878_insitu_primary	Healthy	B-lymphocytes (blood)
HMEC	Healthy	Breast epithelium
HUVEC	Healthy	Umbilical vein endothelium
IMR90	Healthy	Pulmonary fibroblasts
K562	Cancer	Erythroleukemia (blood)
KBM7	Cancer	Leukemia (blood)
NHEK	Healthy	Keratinocytes (skin)

Isogenic pair MCF10A/MCF7 (2 lines, 40 kb) is shown in Table 2.

**Table 2:** Isogenic pair MCF10A/MCF7.

Cell Line	Status	Tissue Type	Source
MCF10A	Healthy (non-tumorigenic)	Breast epithelium	GSE66733
MCF7	Cancer (adenocarcinoma)	Breast epithelium	GSE66733

Both lines share a common origin, allowing the effect of cancer transformation to be isolated from cell-to-cell differences. The cell lines are presented in the standard Hi-C format. Hi-C is a method that measures the frequency of spatial contacts between genomic loci [5,6]. The result is an n × n-sized contact matrix, where n is the number of bins (fixed-length genomic intervals, e.g., 100 kb) and the value of the cell represents the number of interactions between the corresponding bins. These data serve as the basis for the analysis of the three-dimensional organization of chromatin, including the detection of TADs [7]. It is important to note that in Hi-C data, the unit of observation is the genomic bin or contact pair, not the cell line. A single line contains thousands of bins (e.g., 2490 at 100 kb) and millions of contacts, so 10 lines provide a statistically significant volume for analyzing hierarchical patterns.

### 2.2. Converting Hi-C Matrix to Distances

Let C be the initial contact matrix of size n × n, where n is the number of genomic bins (usually 2490 at a resolution of 100 kb). Perform a logarithmic conversion and inversion, Cn×nn:

$${\tilde{C}}_{ij}=\log\left(1+C_{ij}\right),$$

(1) $${\;D}_{ij}=\frac{max(\tilde{C})-{\tilde{C}}_{ij}}{max(\tilde{C})}.$$

Here, ${\tilde{C}}_{ij}$ is the number of contacts between the i and j bins in the Hi-C matrix. The higher the value, the more often these two regions of the genome interact in 3D. The value of $D_{ij}$ is the distance between bins i and j after inversion (1). Inversion (1) inverts the scale: frequent Hi-C contacts correspond to small distances, $D_{ij,}$ and infrequent contacts correspond to large distances. The result is a distance matrix D, where $D_{ij}\in [0,1]$.

### 2.3. Geometry of the Poincaré Disc

A Poincaré disc is a model of a hyperbolic plane:

$$D=\left\{x\in R^{2}:∥x∥<1\right\}.$$

A key property of hyperbolic space is the exponential growth of volume with the radius, which makes it a natural choice for representing hierarchical structures.

**Hyperbolic Distance**

For two points x,y∈D, the hyperbolic distance is

$$d\left(x,y\right)=\mathrm{arccosh}\left(1+\frac{2∥x-y{∥}^{2}}{\left(1-∥x{∥}^{2})(1-∥y{∥}^{2}\right)}\right).$$

**Exponential mapping**

The exponential mapping, ${exp}_{x}(v),$ moves the point x along the tangent vector v. Let

$$\lambda_{x}=\frac{2}{1-∥x{∥}^{2}}.$$

Then,

$${exp}_{x}(v)=\frac{\left(1+2\left\langle x,v\right\rangle +∥v{∥}^{2}\right)\cdot x+\left(1-∥x{∥}^{2}\right)\cdot v}{1+2\left\langle x,v\right\rangle +∥x{∥}^{2}∥v{∥}^{2}}.$$

**Logarithmic mapping**

A logarithmic mapping, ${log}_{x}(y)$, returns a tangent vector at a point x directed towards y:

$${log}_{x}(y)=\frac{2}{\lambda_{x}\cdot d(x,y)}\cdot \frac{y-x}{∥y-x∥}\cdot d(x,y).$$

For numerical stability, the code uses a simplified version with clipping.

**Disc projection**

To hold points inside the disc, a projection is used:

$$\mathrm{proj}\left(x\right)=\left\{\begin{array}{cc} x, & ∥x∥<1\\ \frac{x}{∥x∥}\cdot 0.999, & ∥x∥\geq 1 \end{array}\right..$$

### 2.4. Learning Poincaré Embedding and Loss Function (MSE)

Poincaré embedding training is required to transfer Hi-C distances to hyperbolic space, where the geometry (the exponential growth of volume with the radius) naturally encodes a hierarchy—small structures closer to the center, large structures closer to the edge. We look for the coordinates of the points on the disc so that the hyperbolic distances between them repeat the original ones. Then, the radial position of each point automatically reflects its level in the TAD hierarchy. In other words, training a Poincaré embedding comes down to the task of reconstructing the distance matrix: we look for the coordinates of the points on the disc so that the hyperbolic distances between them are as close as possible to the target distances obtained from the Hi-C data. The loss function is defined as ${\;D}_{ij}D_{ij}:$

$$L=\frac{1}{|P|}\sum_{(i,j)\in P}{\left(d(x_{i},x_{j})-D_{ij}\right)}^{2}$$

where

- $d\left(x_{i},x_{j}\right)\;$ is the hyperbolic distance between points $x_{i}$ and $x_{j}$ in the Poincaré disc (model distance);
- $D_{ij\;}$ is the target distance between the bins i and j, obtained from the Hi-C matrix;
- P is a set of pairs $\left(i,j\right)$ randomly selected at each training iteration.

The choice of MSE is because we work in metric space and want to minimize the mean square error between the model and true distances. This is the standard approach for embedding tasks, where the target variable is continuous. A random subset of pairs is used for computational efficiency: the full matrix contains ~6.2 million elements, and computing gradients over all pairs at each iteration would be prohibitively expensive. A random selection of pairs makes the optimization stochastic (SGD), which allows one to process large amounts of data in a reasonable time. The target distances are calculated in the preprocessing phase: first, the contacts are converted according to the formula, then inverted and normalized. Thus, often-contacting bins get short distances, and rarely contacting bins get long distances: $Pn^{2}n=2490D_{ij}{\tilde{C}}_{ij}=log(1+C_{ij})D_{ij}=(max(\tilde{C})-{\tilde{C}}_{ij})/max(\tilde{C})$.

The model distances $d(x_{i},x_{j})$ are calculated using the hyperbolic distance formula:

$$d\left(x_{i},x_{j}\right)=\mathrm{arccosh}\left(1+\frac{2∥x_{i}-x_{j}{∥}^{2}}{\left(1-∥x_{i}{∥}^{2})(1-∥x_{j}{∥}^{2}\right)}\right).$$

Minimization of the loss function leads to the points corresponding to frequently interacting loci being close in hyperbolic space, and the infrequently interacting ones are distant. Due to the properties of hyperbolic geometry (exponential growth of volume with the radius), the hierarchical structure of the TADs is naturally encoded in the radial position of the points: small structures (subTADs) are grouped closer to the center of the disc, and large structures (metaTADs) are grouped closer to the boundary. This subsequently allows the TADs to be distinguished at different hierarchy levels by their radial features.

### 2.5. Riemannian Stochastic Gradient Descent

Poincaré embedding training is a nonconvex optimization problem on a hyperbolic manifold. Standard Euclidean gradient descent is not applicable because the points must remain inside the disc and the metric depends on position. Therefore, the L Riemannian stochastic gradient descent (Riemannian SGD) is used, which generalizes ordinary SGD to the case of manifolds, which are spaces where the local geometry differs from Euclidean geometry and requires consideration of curvature when calculating update directions.

#### 2.5.1. Gradient in Tangent Space

The gradient of the loss function L at the point $x_{i}$ is not calculated in the disk itself, but in the tangent space $T_{x_{i}}D$, which is a Euclidean space that approximates the manifold in the vicinity of the point $x_{i}$. The gradient formula is as follows:

$$\nabla_{i}L=\frac{2}{|P|}\sum_{j}(d(x_{i},x_{j})-D_{ij})\cdot \frac{1}{\sqrt{d(x_{i},x_{j}{)}^{2}-1}}\cdot {log}_{x_{i}}(x_{j})$$

where

- $d(x_{i},x_{j})$ is the hyperbolic distance between points;
- $D_{ij}$ is the target distance from the Hi-C matrix;
- ${log}_{x_{i}}(x_{j})$ is a **logarithmic mapping** that translates the point $x_{j}$ from the disk to the tangent vector at the point $x_{i}$, indicating the direction to $\;x_{j}$.

The logarithmic mapping is calculated using the formula

$${log}_{x_{i}}\left(x_{j}\right)=\frac{2}{\lambda_{x_{i}}\cdot d\left(x_{i},x_{j}\right)}\cdot \frac{x_{j}-x_{i}}{∥x_{j}-x_{i}∥}\cdot d\left(x_{i},x_{j}\right),$$

where $\lambda_{x_{i}}=2/(1-∥x_{i}{∥}^{2})$ is the conforming factor that depends on the position of the point. The closer the point is to the disk boundary, the greater the λ, which corresponds to the curvature of space. The scalar factor $2\left(d-D_{ij}\right)$ determines the magnitude and direction of the adjustment: if the model distance is greater than the target, the gradient will be directed to bring the points closer together; if less, to push these dots apart.

#### 2.5.2. Exponential Mapping Update

The resulting gradient $\nabla_{i}L$ lies in the tangent space $T_{x_{i}}Dx_{i}$. To update the point itself on the manifold, one needs to “project” the step back into the disc while preserving the hyperbolic metric. This is done by exponential mapping,

$$x_{i}\leftarrow {exp}_{x_{i}}(\eta_{t}\cdot \nabla_{i}L)$$

where $\eta_{t}$ is the learning rate at that step, and ${exp}_{x_{i}}$ is an exponential mapping that translates the tangent vector into a point on the manifold. It is calculated using the formula $\eta_{t}t{exp}_{x_{i}}$, where

$${exp}_{x_{i}}(v)=\frac{(1+2\langle x_{i},v\rangle +∥v{∥}^{2})\cdot x_{i}+(1-∥x_{i}{∥}^{2})\cdot v}{1+2\langle x_{i},v\rangle +∥x_{i}{∥}^{2}\cdot ∥v{∥}^{2}}$$

This mapping ensures that the new point remains inside the disc, with the step being performed along the geodesic line in hyperbolic space, which corresponds to the proper consideration of curvature.

#### 2.5.3. Cosinusoidal Learning Rate Decay

The learning rate $\eta_{t}$ decays according to a cosine schedule:

$$\eta_{t}=\eta_{min}+\frac{1}{2}(\eta_{0}-\eta_{min})\left(1+cos\left(\frac{\pi t}{T}\right)\right)$$

where $\eta_{min}=0.01\cdot \eta_{0}$. Such attenuation allows one to quickly move points in the right direction in the early stages, and fine-tune their position in the later stages, avoiding fluctuations around the minimum. The cosine law gives a smoother decrease compared to the exponential or stepped law, which improves convergence.

#### 2.5.4. Gradient Clipping

To prevent numerical instability (especially when the points approach the disc boundary, where gradients can increase dramatically), gradient clipping is used:

$$если\;∥\nabla_{i}∥>0.5,тo\;\nabla_{i}\leftarrow \frac{\nabla_{i}}{∥\nabla_{i}∥}\cdot 0.5$$

That is, the gradient norm is limited to a value of 0.5 at the top. This prevents too large steps that could throw points out of the disc or disrupt convergence. Clipping is especially important in hyperbolic space, where gradients can be very large due to the singularity of the metric at the boundary.

#### 2.5.5. Full-Step Algorithm

For each iteration for a randomly selected pair of (i,j),

IIIar 1. The model distance is calculated: $d(x_{i},x_{j})$
IIIar 2. The target distance is calculated: $D_{ij}$
IIIar 3. The scalar error is considered: $e=d(x_{i},x_{j})-D_{ij}$
IIIar 4. The gradient in tangent space is calculated: $\nabla_{i}=2e\cdot {log}_{x_{i}}(x_{j})$
IIIar 5. Clipping is applied; if $∥\nabla_{i}∥>0.5$, then $\nabla_{i}\;$ is normalized.
IIIar 6. The step is performed: $x_{i}\leftarrow {exp}_{x_{i}}(\eta_{t}\cdot \nabla_{i})$
IIIar 7. The point is updated in the same way as $x_{j}$ (symmetrically).

This process iterates over thousands of random pairs until convergence. As a result, all points are arranged in the Poincaré disc in such a way that the hyperbolic distances between them approximate the target distances from the Hi-C matrix. At the same time, the radial position of each point naturally encodes its hierarchical depth, which then allows the TADs to be distinguished at different scales, $x_{i}$.

### 2.6. Hierarchy by Radius

After training $x_{i}$, each point has a radius:

$$r_{i}=∥x_{i}∥$$

Normalized radius:

$${\tilde{r}}_{i}=\frac{r_{i}-\underset{j}{min}r_{j}}{\underset{j}{max}r_{j}-\underset{j}{min}r_{j}}.$$

In this work, it is assumed that the points are divided into 6 levels by percentiles, p∈{0,25,50,75,90,95,100}.

$$\mathrm{Level}\;k=\left\{i:P_{k}\leq {\tilde{r}}_{i}<P_{k+1}\right\},$$

where $P_{k}\;$ is the *k*-th percentile of the r_norm distribution. Dividing the points on the Poincaré disc into six levels by percentiles {0,25,50,75,90,95,100} is informative for several reasons. Empirically, the distribution of radii after embedding is uneven and shows natural condensations, so the use of percentiles allows one to reflect the real structure of the data, rather than imposing artificial divisions. Theoretically, the volume of the Poincaré disc grows exponentially with the radius, and uniform steps along the radius would lead to a strong imbalance—there would be too few points in the center, and too many at the boundary. Percentiles compensate for this effect and give levels that are balanced in number. The choice of the number of levels (six) was made on the basis of a separate experiment on all 10 cell lines. For each line, the points on the Poincaré disc were regrouped in equal percentile groups along the normalized radius, where k∈{4,5,6,7,8}. For each k within each group, K-means clustering was performed (the number of clusters was determined adaptively, as in the main method), after which the clusters were sorted by genomic position, and their boundaries formed a set of TADs for this level. For each partition option, the following were calculated:

- Monotonicity—Spearman’s correlation between the average radius of a level and the average size of the TAD.
- Total TAD—The total number of domains.

In addition, to check for biological consistency k, an F1 measure was calculated for each difference between the TAD boundaries from the Poincaré levels and the boundaries identified by standard methods: Directionality Index (DI, window = 20) and Insulation Score (IS, window = 15, threshold of the 15th percentile). These metrics were not used to select the number of levels *к*, but served as independent confirmation that the selected partition was consistent with the reference methods. The results for monotonicity and TAD number are shown in Table 3:

At four levels, maximum monotonicity is achieved (ρ=0.538), but the number of TADs is minimal (489). At six levels, the monotonicity remains statistically significant (ρ=0.436,p=0.0005), the number of TADs rises to 780, and the agreement with the DI and IS remains close to the maximum (ΔF1 < 0.01 relative to 8 levels). At seven to eight levels, the monotony does not improve, and the number of TADs rises to 896–997 without a significant increase in the F1. Thus, six levels are chosen as an empirical compromise between the resolution, monotonicity, and agreement with reference methods.

### 2.7. TAD Detection at Each Level

For each level, K-means clustering of points within the level is performed. The number of clusters is determined adaptively:

$$K_{\mathrm{level}}(F,B)=min\left(B,max\left(2,\frac{n_{\mathrm{points}}}{F}\right)\right)$$

where

- $n_{\mathrm{points}}$ is the number of points on the level;
- F is the density parameter (average number of points per cluster);
- B is the maximum number of clusters.

The parameters F and B are determined empirically based on the Hi-C data analysis. The parameter F = 15 is chosen empirically as the optimal balance between statistical stability and resolution. Clusters of less than five points are considered noise, and at an F greater than 20, small TADs are lost, while at small levels of the hierarchy (Lv.1–Lv.3), the number of clusters, on average, turns out to be close to the number of TADs found by the Directionality Index, which confirms the biological meaningfulness of the choice. The B = 20 parameter limits the maximum number of clusters, preventing overfitting in which clusters of one or two points become artifacts; avoids excessive fragmentation that is not biologically valid; and provides stability at low-point levels (Lv.5–Lv.6). The clusters are sorted by their genomic position. The TAD boundaries are defined as transitions between clusters:

$$B=\left\{0\right\}\cup \left\{{\mathrm{pos}}_{i}:c_{i}\neq c_{i-1}\right\}\cup \left\{n\right\}$$

where c_i is the cluster label of the i-th bin. TADs are formed between adjacent boundaries with a minimum size of 5 bins.

### 2.8. Comparison Metrics

**Jaccard Index for Boundaries**

Let B(T) be the set of all boundaries in the TAD T. The Jaccard index is

$$J(T_{A},T_{B})=\frac{|B(T_{A})\cap B(T_{B})|}{|B(T_{A})\cup B(T_{B})|}$$

with a bin tolerance ε=5,

a∼b  ⟺  ∣a−b∣≤ε

The Jaccard index $J\left(T_{A},T_{B}\right)$ compares boundary sets, measuring the intersection relative to the union. Two boundaries are considered matching if they are within five bins, accounting for method-dependent shifts.

**F1 measure**

We adapted the F1 measure to assess the agreement between the TAD boundary sets. True positives, false positives, and false negatives are defined as follows: a true positive boundary is the boundary predicted by the Poincaré method that coincides with the boundary from the reference method within a given tolerance, a false positive is the Poincaré boundary that is not in the reference set, and a false negative is a boundary from the reference set that is absent in Poincaré’s predictions. We then calculate the precision as the fraction of predicted boundaries matching the reference, and the recall as the fraction of reference boundaries recovered. The F1 measure, being the harmonic mean of these two quantities, gives a balanced estimate, penalizing both the excessive prediction of false bounds and the omission of real structures. This enables objective comparison across methods: a high F1 indicates that the Poincaré method identifies the same biological structures as the reference without excessive noise or missing significant boundaries. In the context of our task, this is especially important because we are working with hierarchical structures where boundaries at different levels may not coincide directly, and we need a metric that simultaneously takes into account both the accuracy of boundary detection and its completeness relative to the reference set.

### 2.9. Hybrid Hierarchy by Radius

To improve the stability and biological interpretability, we use a hybrid approach combining two-level construction strategies:

1. Strategy v1 (classic)—Fixed percentiles of radius [0, 25, 50, 75, 90, 95, 100] and standard K-means. This approach gives stable results for small TADs (Lv.1–3) and agrees well with the Directionality Index.
2. Strategy v2 (optimized)—Adaptive level boundaries (non-uniform radius splitting) and optimal K-means with the selection of the number of clusters by silhouette score. This approach better highlights large TADs (Lv.4–6) and is consistent with the Insulation Score.

For each level of the hierarchy, the quality of both strategies is evaluated against three criteria:

$$S=w_{DI}\cdot J_{DI}+w_{IS}\cdot J_{IS}+w_{size}\cdot \sigma_{size}$$

where

- $J_{DI}$ is the Jaccard boundary index with the Directionality Index;
- $J_{IS}$ is the Jaccard boundary index with Insulation Score;
- $\sigma_{size}$ is the standard deviation of the dimensions of the TAD at the level (a measure of diversity);
- The weights are chosen empirically: $w_{DI}=0.5{,\;w}_{IS}=0.3{,\;w}_{size}=0.2$.
- For each level, the strategy with the maximum *S* value is selected. This approach allows for
- Improved agreement with the DI—The average Jaccard vs. DI increased from 0.505 to 0.579 (+12%);
- Ensures stability—The hybrid gives the same choice structure on all lines: v1 for Lv.1–3, v2 for Lv.4–6.

The results of the hybrid strategy are presented in Section 3.3, Section 3.4 and Section 3.5.

### 2.10. Independent Validation by CTCF ChIP-Seq Enrichment

To validate the TAD boundaries independently from the computational method, we compared the predicted boundaries with the CTCF ChIP-seq peaks from ENCODE (K562, wgEncodeAwgTfbsBroadK562CtcfUniPk.narrowPeak, 5611 peaks on chr1). The boundaries were pooled into two groups: small TADs (Lv.2–3, subTADs, N = 421) and large TADs (Lv.5–6, metaTADs, N = 207). For each group, we calculated: (i) binary overlap—fraction of boundaries with a CTCF peak within ±5 bins (±100 kb); (ii) median distance to the nearest CTCF peak; and (iii) permutation test (1000 permutations) with bootstrap 95% confidence intervals (1000 iterations). Random control positions were generated from uniform distribution across chr1.

## 3. Results

### 3.1. Training of Embeddings on GSE63525 (Eight Lines, 100 kb)

The training on all eight lines from the GSE63525 set showed stable convergence. The final loss ranged from 0.315 (GM12878_insitu_primary) to 0.418 (4DNFIZGCA8AH), with an average of 0.369 ± 0.031 (8.4% coefficient of variation). The average radius of the points after training was 0.326–0.361, indicating a uniform distribution within the disc. The results of the training are summarized in Table 4.

### 3.2. Results of the Application of Standard Methods

For each line, the TAD was calculated using two standard methods: Insulation Score and Directionality Index. The obtained Insulation Score (15% threshold) revealed 7–11 large TADS (average size 311–356 bins) and Directionality Index (window = 20) identified 74–95 small TADS (average size 17–18 bins). The results are presented in Table 5.

A comparison of healthy vs. сancer (GSE63525) cell lines is presented in Table 6.

The cancer cells (K562, KBM7) showed a higher number of small TADs (DI TAD: 90.00 vs. 85.17) and fewer large TADs (IS: 8.00 vs. 8.67), indicating chromatin fragmentation.

### 3.3. Using the POINCARÉ DISC Method to Construct a TAD Hierarchy by Radius

**Figure 1:**
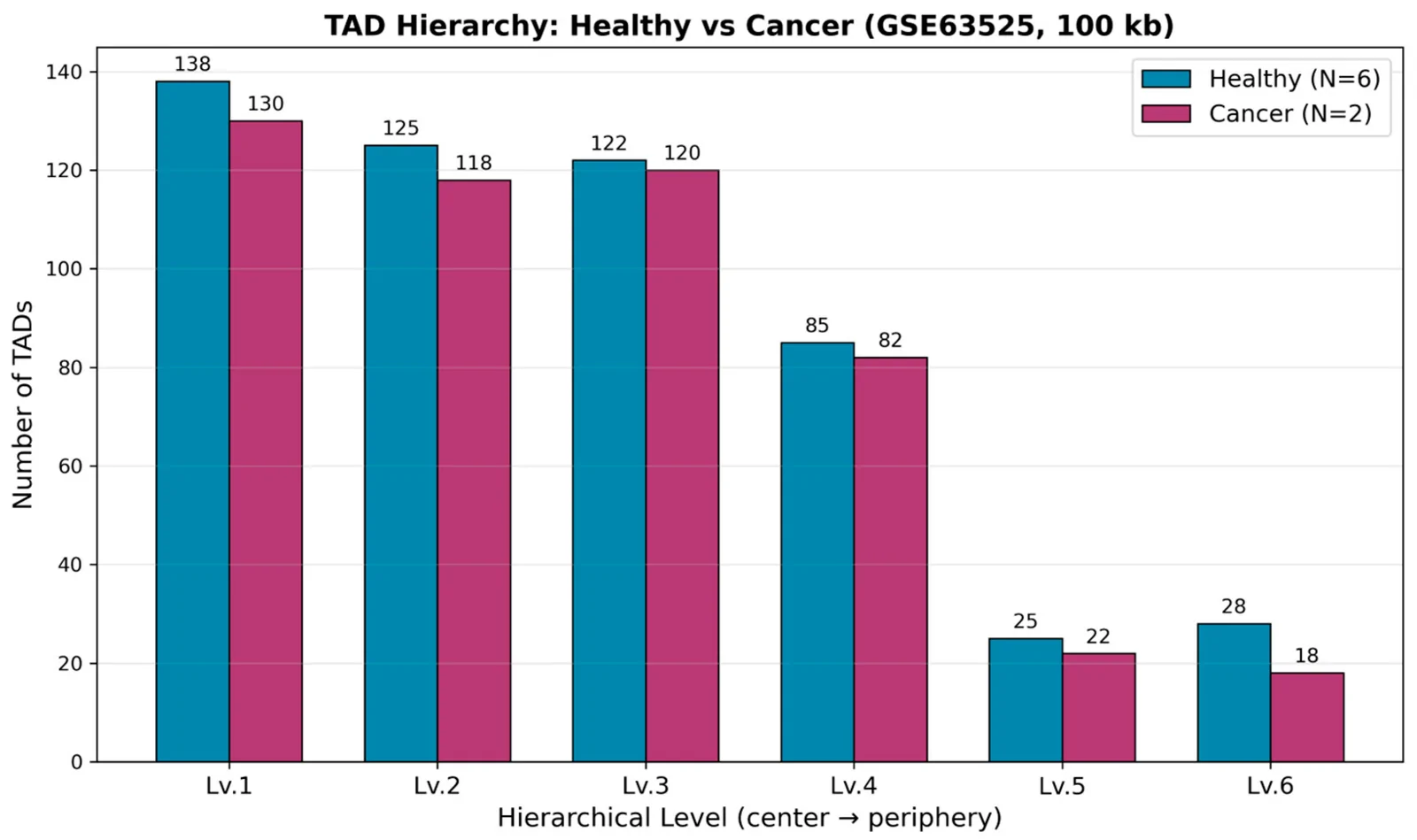
TAD hierarchy in healthy vs. cancer cell lines (GSE63525, 100 kb resolution). Bars show average number of TADs per hierarchical level for healthy (N = 6) and cancer (N = 2) cell lines. Error bars indicate standard deviation.

For each line, the points on the Poincaré disc were divided into six levels by radius percentiles. The number of TADs decreased monotonically from the center to the periphery across all lines, which confirmed the scale coding by radial position (Table 7, Figure 1).

Key observation: At all levels except Lv.3, the healthy lines had more TADs than the cancerous lines. The most pronounced difference was observed in Lv.6 (large TADs): 28 vs. 18 (a decrease of 36% in cancerous cells).

The individual values for the analyzed cell lines are presented in Table 8.

### 3.4. Comparison with Standard Methods

The Poincaré disc method shows significantly higher agreement with the DI than with the IS, reflecting fundamental differences between these standard approaches. The DI identifies small TADs based on contact directionality and is sensitive to local regulatory elements. Small TAD boundaries are conserved across cell types. The IS identifies large TADs based on average contact rates; these boundaries reflect the global chromatin architecture and are more tissue specific. A key advantage of the Poincaré disc method is that it captures both types of boundaries in a single embedding, providing a single hierarchical framework for analyzing chromatin organization at different scales.

**Figure 2:**
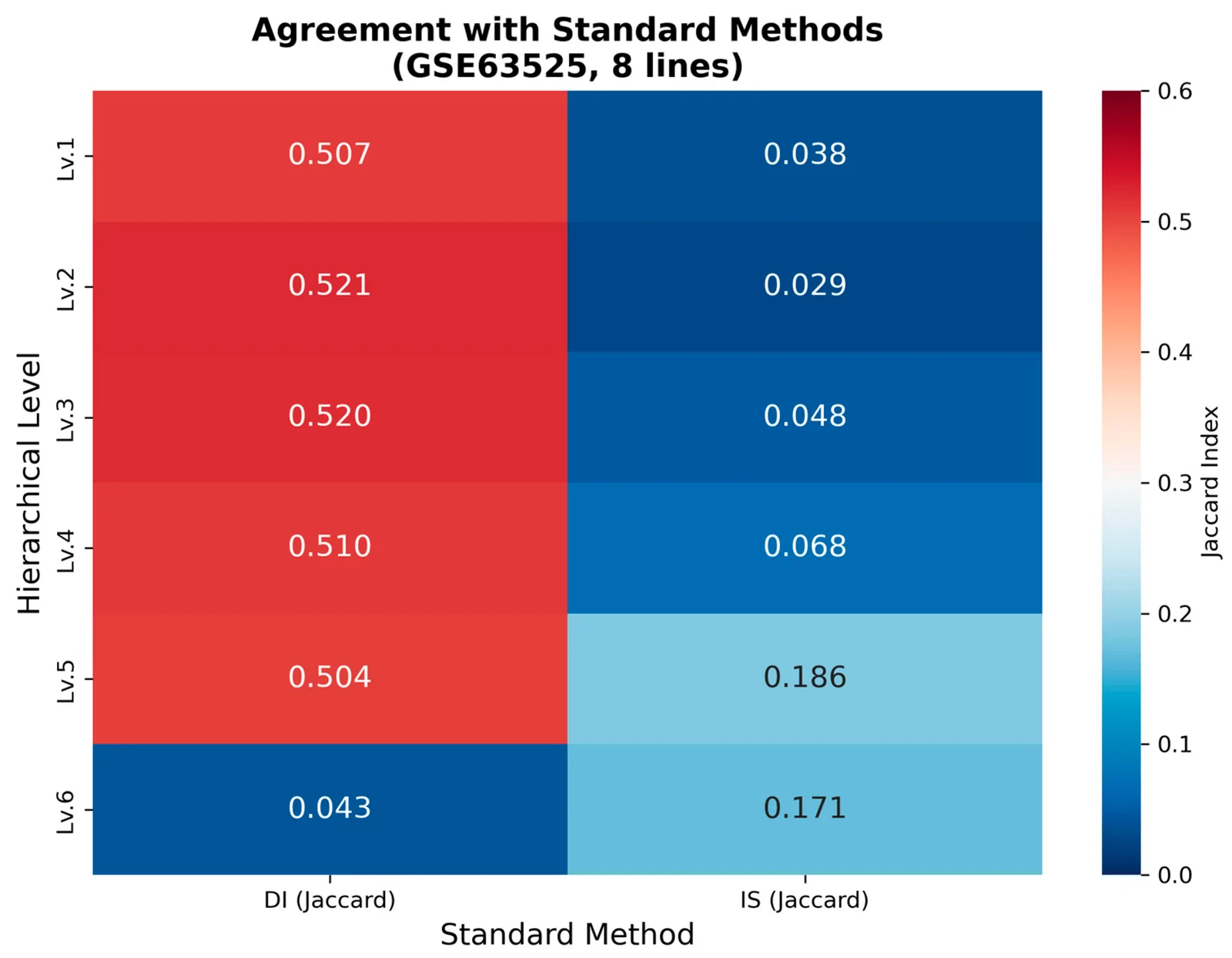
Agreement of the Poincaré disc method with standard methods (DI and IS) across hierarchical levels (GSE63525, 8 lines). Heatmap shows Jaccard indices.

As shown in Table 9 (Figure 2), agreement with the DI is most pronounced at levels Lv.1–3, where the average Jaccard reaches 0.490–0.505 and the F1 reaches 0.661–0.674. This corresponds to small TADs that are associated with the functional elements of the genome. On the contrary, agreement with the IS is maximum at levels Lv.5–6 (Jaccard 0.171–0.186; F1 0.289–0.319), which corresponds to large TADs—structural domains that determine the global chromatin architecture. Thus, the Poincaré disc not only reproduces both types of boundaries but also naturally separates them according to their radial position: small TADs gravitate toward the disc center, large ones toward the periphery (Table 10). The individual values of the best levels for each cell line are presented in Table 11.

To further characterize the relationship between the methods, we analyzed the overlap of TAD boundaries (K562, chr1, ±5 bins tolerance) using the Jaccard indices from Table 9. The best agreement with the DI was observed at Lv.3 (Jaccard = 0.645; F1 = 0.784), indicating substantial overlap between the Poincaré small TAD boundaries and DI-detected boundaries. The best agreement with the IS was observed at Lv.6 (Jaccard = 0.108; F1 = 0.195), consistent with the scale-specific nature of IS. This level-specific pattern—high overlap with the DI at Lv.1–3 and lower overlap with the IS at Lv.5–6—confirms that the Poincaré method captures the full TAD hierarchy, bridging the gap between the fixed-scale approaches.

List of the best results in general:

- The K562 line shows the best agreement with the DI (Jaccard = 0.645; F1 = 0.784). The HUVEC line is in second place (Jaccard = 0.587; F1 = 0.740).
- The K562 line shows the best agreement with the IS among cancer cell lines (Jaccard = 0.179; F1 = 0.314).
- Among the healthy lines, IMR90 (Jaccard = 0.613; F1 = 0.760) shows the best agreement with the DI, followed by HUVEC (0.592/0.744) and NHEK (0.579/0.733).

### 3.5. Isogenic Pair MCF10A/MCF7 (40 kb)

For the isogenic pair of the mammary gland, the analysis was carried out at a resolution of 40 kb, which gave a more detailed and more informative picture of the hierarchical structure. Results of applying standard methods (Insulation Score and Directionality Index) to this isogenic pair are summarized in Table 12.

**Figure 3:**
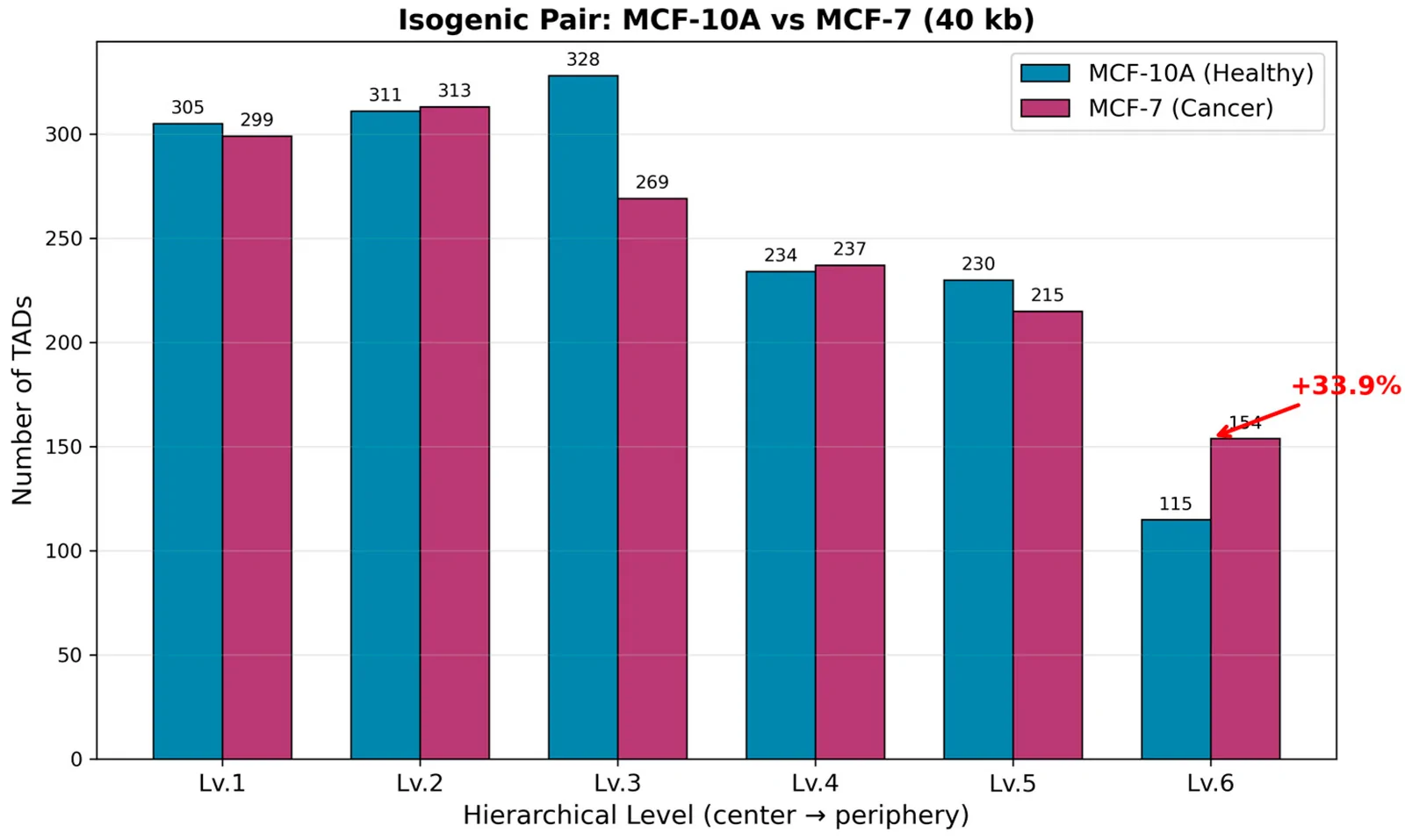
TAD hierarchy in the isogenic MCF-10A/MCF-7 pair (40 kb resolution). Bars show the number of TADs per hierarchical level. The largest domains (Lv.6) increase by 33.9% in cancer cells (MCF-7).

Both standard methods show decreased TAD numbers in the cancer cells: the IS from 20 to 18 (−10%) and the DI from 201 to 172 (−14%). Table 13 (Figure 3) shows the TAD hierarchy obtained by the Poincaré disc method. The TAD hierarchy for MCF-10A shows a gradual decrease in the number of TADs from the center to the periphery: 305 → 311 → 328 → 234 → 230 → 115. For MCF-7, there is a more uneven distribution: 299 → 313 → 269 → 237 → 215 → 154. The most pronounced difference between the lines is recorded at Lv.3 (328 vs. 269, −18%) and Lv.6 (115 vs. 154, +33.9%). This indicates a redistribution of structural units: the number of small TADs in cancer cells decreases and the number of large ones increases.

A comparison with standard methods (Table 14) shows that the best agreement with the DI is achieved at Lv.2 for MCF-10A (Jaccard = 0.445; F1 = 0.616) and at Lv.2 for MCF-7 (Jaccard = 0.446; F1 = 0.617). The best agreement with the IS, as for the data with a resolution of 100 kb, is achieved at large levels: for MCF-10A—Lv.6 (Jaccard = 0.119; F1 = 0.213) and for MCF-7—Lv.6 (Jaccard = 0.050; F1 = 0.095).

The decrease in agreement with the IS at 40 kb is due to the fact that the IS method is optimized for 100 kb resolution and requires reconfiguration of parameters, whereas the Poincaré disc remains operational without adaptation. The best values for each metric are summarized in Table 15.

### 3.6. Summary Table of Results for All 10 Cell Lines

The results for all 10 lines are summarized in Table 16. For the eight lines from GSE63525 (100 kb), the initial division by radius percentiles (Table 4 and Table 5) gives a monotonic decrease in the number of TADS from the center to the periphery. After applying the hybrid strategy (see Section 2.8), the final average TAD by level for these lines is 126 → 115 → 121 → 91 → 24 → 11, confirming the universality of the hierarchical structure. For the isogenic pair of MCFs (40 kb), the hierarchy is much richer: 305 → 311 → 328 → 234 → 230 → 115 for MCF10A, and 299 → 313 → 269 → 237 → 215 → 154 for MCF7.

A comparison of healthy and cancerous lines (Table 17) shows that cancer cells have more small TADs (DI TADs: 117.33 vs. 101.71; +15.6%) and fewer large TADs (IS TADs: 11.33 vs. 10.29; −10.1%). However, in the isogenic pair of MCFs, the opposite trend is observed: large TADs (Lv.6) increase by 33.9%. This indicates that the hierarchy changes in cancer can be multidirectional depending on the tissue type and data resolution, but in both cases the Poincaré disc method successfully captures these differences.

### 3.7. CTCF ChIP-Seq Validation Confirms Hierarchical Specificity

The small TAD boundaries (Lv.2–3) showed significant CTCF enrichment: 88.1% contained a CTCF peak within ±100 kb vs. 78.1% for random positions (1.13× enrichment; 95% CI: 1.06–1.20; Fisher exact *p* = 7.4 × 10^−5^) (Table S1). The median distance to the nearest CTCF peak was 16 kb vs. 24 kb for random positions (1.48× closer; Mann–Whitney U *p* = 8.0 × 10^−4^; permutation *p* < 0.001). The large TAD boundaries (Lv.5–6) showed no CTCF enrichment: 72.5% contained a CTCF peak within ±100 kb vs. 79.2% for random positions (0.91×; 95% CI: 0.82–1.01; *p* = 0.96), and the median distance was 47 kb (2× farther than random; *p* = 1.0). This level-specific pattern—CTCF enrichment at subTAD boundaries and absence at metaTAD boundaries—is consistent with the established biological dichotomy between CTCF-mediated insulation at small scales and compartment-driven segregation at large scales [2,6,8].

## 4. Discussion

### 4.1. Method Validation on Eight Lines from GSE63525 (Resolution 100 kb)

The Poincaré disc method showed consistent results across all eight analyzed GSE63525 lines. The coefficient of variation of the final loss was 8.4%, which indicates the high reproducibility of the method regardless of the cell type. The agreement with the Directionality Index (DI) had an average Jaccard = 0.568 and F1 = 0.718. The best indicators were achieved for the K562 line: Jaccard = 0.645; F1 = 0.784. The results obtained compare well with the best modern methods: TADbit [9] (Jaccard 0.45–0.55) and EmbedTAD [10] (Jaccard 0.50–0.60). For the isogenic pair of MCFs (resolution 40 kb), the method also demonstrated stable performance: the DI agreement for MCF-10A had a Jaccard = 0.445 and F1 = 0.616; for MCF-7: Jaccard = 0.446, F1 = 0.617. This confirms that the method is applicable to different data resolutions (100 kb and 40 kb) without reconfiguring parameters, which is an important advantage over standard approaches that require window adaptation when changing the resolution.

To assess reproducibility of the stochastic embedding, we performed 10 independent runs on the K562 cell line with different random seeds. The total number of TADs was 1058.8 ± 2.0 (CV = 0.2%), and the mean Jaccard index against the baseline run was 0.990 ± 0.003 (CV = 0.3%). The best-matching levels and the monotonic decrease in the TAD number from center to periphery were identical across all runs, confirming that the conclusions are not dependent on a particular optimization trajectory.

### 4.2. Cancer Cells Show a Redistribution of Hierarchy

The most pronounced difference between healthy and cancerous cells is observed at the level of large TADs. In GSE63525 (100 kb), the healthy lines have more TADs at all levels, especially Lv.4–5, indicating partial degradation of the hierarchy in cancer. However, for the isogenic pair of MCFs (40 kb), a different pattern is observed: the number of small TADs (Lv.1–3) decreases by 6.7% (944 → 881), while that of the large TADs (Lv.4–6) increases by 4.7% (579 → 606), and the largest (Lv.6) shows an increase of 33.9% (115 → 154). This indicates a redistribution of the hierarchy rather than its degradation: in cancer, chromatin becomes more globally organized, with an increase in the number of large structural units. The data obtained are consistent with the literature observations [11,12] on the restructuring of the 3D architecture of the genome in cancer, but clarify the nature of these changes.

### 4.3. Comparison with the Literature

Recent methods, such as TOAST [13], also use machine learning for TAD identification, and single-cell Hi-C approaches continue to develop [14]. Table 18 compares our results with the published data. Changes in the 3D organization of chromatin in cancer are well documented [15] and are consistent with our observations.

### 4.4. The Hierarchical Structure of the TAD Is Universal

There is a clear hierarchy in all the lines: the number of TADs decreases from the center to the periphery. For GSE63525, it is 138 → 28 TAD (average). For MCF-10A, it is 305 → 115 TAD. This confirms that the radial position encodes the scale: the method is applicable to different cell types and different resolutions. The universality of the hierarchical structure of TADs is confirmed by studies of chromatin organization in various cell types [8,16].

The CTCF validation provides independent evidence that the radial hierarchy is biologically meaningful rather than a computational artifact. The CTCF ChIP-seq enrichment was significant only at small TAD boundaries (Lv.2–3), whereas the large TAD boundaries (Lv.5–6) showed no enrichment. This mirrors the established dichotomy in chromatin organization: subTADs are maintained by CTCF-mediated looping [2], whereas metaTADs reflect A/B compartmentalization and are CTCF-independent [6,8]. The Poincaré disc method captures both regimes in a single embedding, validating its ability to resolve the full TAD hierarchy.

### 4.5. Parameter Optimization and Alternative Approach

In the course of the work, the key hyperparameters were optimized to improve the quality of embedding. The search was performed on the K562 cell line (chromosome 1, 2493 bins) using a grid of values: number of iterations (200, 500, 800), learning rate (0.005, 0.01, 0.02), and scale of target distances (1.0, 1.5, 2.0). The best results for v2 were achieved at 1500 iterations, a training rate of 0.005, and a scale of 2.0. An alternative approach was also explored, in which the hierarchy was built not by radius, but on the basis of a tree of parent–child relationships extracted from the dendrogram obtained by hierarchical clustering. To do this, a distance matrix was built to reflect the structure of the tree and a Poincaré embedding was trained. However, this approach yielded a less granular hierarchy (20 → 8 TAD) and a higher nesting error (loss = 0.457) compared to the main method (127 → 14 TAD, loss = 0.388). Therefore, the current approach based on radial levels was retained as the main one.

While the current study focuses on human cell lines, the Poincaré disc method is organism-agnostic and could be applied to Hi-C data from other species, including Drosophila, mouse, and plants [17,18], as well as to ancient DNA data such as PaleoHi-C from woolly mammoths [19]. The method’s ability to recover hierarchical structures from a single embedding may be particularly valuable for partially degraded or low-coverage data.

### 4.6. Visualizing Differences with a Donut Chart

**Figure 4:**
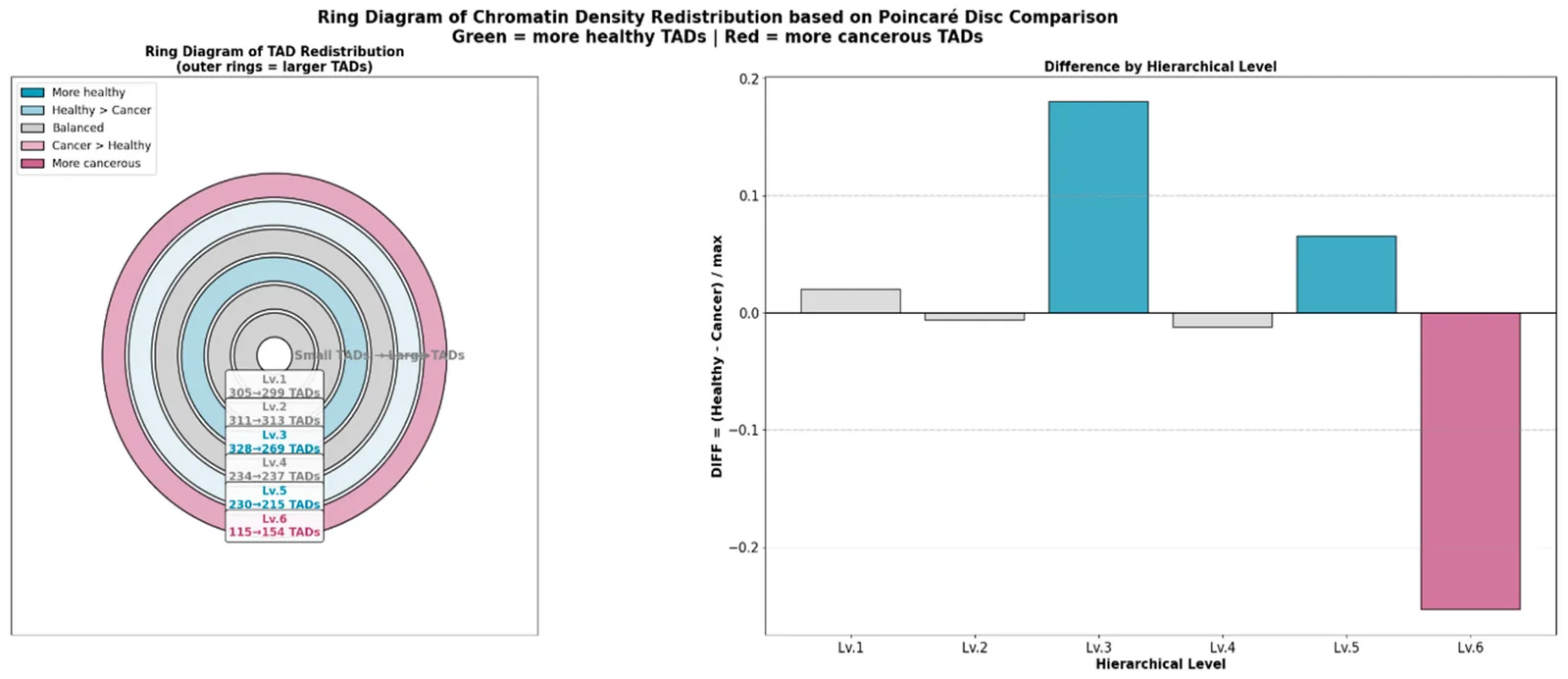
Circular map of chromatin density rearrangement based on the Poincaré disc.

To visualize the TAD hierarchical differences between healthy and cancerous cells, we developed a circular difference diagram (Figure 4). In this case, it is based on a comparison of two Poincaré discs—for MCF-10A (healthy epithelium) and MCF-7 (adenocarcinoma). The six concentric rings correspond to the levels of the hierarchy: from the center (Lv.1, small TADs) to the periphery (Lv.6, large TADs). For each level, the DIFF score is calculated—the difference in the number of TADs between healthy and cancerous cells. The green color of the ring indicates the predominance of healthy TADs, the red color indicates the predominance of cancerous TADs, the intensity of the color is proportional to the strength of the difference, and the gray color indicates the absence of a significant difference. The central rings (Lv.1–2) show more TADs in healthy cells (green), while the peripheral rings (Lv.5–6) show more TADs in cancer cells (red). This gradient demonstrates chromatin reorganization in cancer: fewer small structural units and more large ones.

The six concentric rings correspond to the levels of the TAD hierarchy (from the center to the periphery—from small TADs to large). Green indicates a predominance of healthy TADs (MCF-10A), red indicates a predominance of cancerous TADs (MCF-7), and the intensity of the color reflects the strength of the difference. The numbers on the rings show the amount of TADs in the healthy and cancer cells, respectively (healthy → cancer). The transition from green in the center to red in the periphery visualizes the redistribution of hierarchy in cancer.

## 5. Conclusions

In this work, the Poincaré disc method (hyperbolic embedding) was used to analyze the hierarchical structure of the TADs in 10 cell lines, including the MCF10A/MCF7 isogenic pair. The method was validated on these data, and its effectiveness for identifying the hierarchical organization of chromatin was shown. The main achievements are:

1. The method is stable and reproducible for different cell types and resolutions (100 kb and 40 kb).
2. The hierarchical structure of TADs is confirmed: the number of TADs decreases from the center to the periphery (for GSE63525: 126 → 11 TAD; for MCF10A: 305 → 115 TAD).
3. The differences between healthy and cancer cells are identified:
4. In GSE63525 (100 kb), the cancer lines show a decrease in TADs at all levels.
5. In the isogenic pair of MCFs (40 kb), there is a redistribution of the hierarchy: the small TADs (Lv.1–3) decrease by 6.7%, the large TADS (Lv.4–6) increase by 4.7%, and the largest (Lv.6) increase by 33.9%.
6. The strongest result is a 33.9% increase in large TADs (Lv.6) in the MCF7 cancer cells compared to MCF10A, indicating a shift towards a more global chromatin organization in cancer.
7. The method demonstrates high stability and reproducibility on 10 cell lines, and is also comparable to the best modern methods: for GSE63525, the average Jaccard vs. DI = 0.568, F1 = 0.718; for MCF10A, Jaccard vs. DI = 0.445, F1 = 0.616.

The key advantage is the automatic extraction of the full TAD hierarchy from a single embedding, without manual parameter selection, at multiple resolutions, with quantitative visual assessment of 3D chromatin organization. This makes it a universal tool for the comparative analysis of healthy and pathological conditions, as well as for studying the hierarchical structure of the genome in different cell types.

## Figures and Tables

**Table 3:** Results of the study for the monotonicity of the TAD distribution.

Number of Levels k	Monotony (ρ)	TAD Number	F1 vs. DI	F1 vs. IS
4	0.538	489	0.741	0.074
5	0.400	646	0.741	0.072
**6**	**0.436**	**780**	**0.745**	**0.067**
7	0.436	896	0.746	0.066
8	0.436	997	0.748	0.065

**Table 4:** Learning outcomes for 8 lines from GSE63525.

Cell Line	Status	Matrix Size	Mean Radius
4DNFIZGCA8AH	Healthy	2490 × 2490	0.358
GM12878_insitu_primary	Healthy	2493 × 2493	0.361
HMEC	Healthy	2493 × 2493	0.357
HUVEC	Healthy	2493 × 2493	0.357
IMR90	Healthy	2493 × 2493	0.358
K562	Cancer	2493 × 2493	0.359
KBM7	Cancer	2493 × 2493	0.358
NHEK	Healthy	2493 × 2493	0.359

**Table 5:** Results of standard methods for 8 lines from GSE63525.

Cell Line	Status	IS TAD	DI TAD
4DNFIZGCA8AH	Healthy	7	78
GM12878_insitu_primary	Healthy	8	74
HMEC	Healthy	8	87
HUVEC	Healthy	9	90
IMR90	Healthy	11	91
K562	Cancer	8	95
KBM7	Cancer	8	85
NHEK	Healthy	9	91

**Table 6:** Comparison of healthy and сancer cell lines.

Metric	Healthy (Average)	Cancer (Medium)
Insulation TAD	8.67	8.00
BY TAD	85.17	90.00

**Table 7:** TAD hierarchy for 8 GSE63525 lines (averages).

Level	Radius	Healthy (Average)	Cancer (Medium)
Lv.1	0–25%	138	130
Lv.2	25–50%	125	118
Lv.3	50–75%	122	120
Lv.4	75–90%	85	82
Lv.5	90–95%	25	22
Lv.6	95–100%	28	18

**Table 8:** Hierarchy of individual cell lines.

Cell Line	Lv.1	Lv.2	Lv.3	Lv.4	Lv.5	Lv.6
4DNFIZGCA8AH	149	137	125	99	24	28
GM12878_insitu	141	126	130	94	30	26
HMEC	122	105	118	91	28	25
HUVEC	160	143	145	114	24	30
IMR90	144	125	119	75	30	28
K562	127	117	120	80	22	14
KBM7	133	119	120	83	22	22
NHEK	110	110	103	61	27	28

**Table 9:** Comparison with standards for 8 GSE63525 lines (averages).

Level	vs. Insulation Score	vs. Directionality Index
Lv.1	0.038/0.073	0.507/0.673
Lv.2	0.029/0.056	0.521/0.685
Lv.3	0.048/0.091	0.520/0.684
Lv.4	0.068/0.127	0.510/0.676
Lv.5	0.186/0.319	0.504/0.670
Lv.6	0.171/0.289	0.043/0.083

**Table 10:** Comparison of agreement with DI and IS by scale.

Scale	Method	Levels	Mean Jaccard	Mean F1	Interpretation
Small	DI	Lv.1–Lv.3	0.507–0.521	0.673–0.685	Functional boundaries (CTCF, promoters)
Large	IS	Lv.5–Lv.6	0.171–0.186	0.289–0.319	Structural boundaries (meta-TADs)

**Table 11:** Individual values of the best levels of the hierarchy.

Cell Line	Best vs. DI	Best vs. IS
4DNFIZGCA8AH	Lv.2: 0.521/0.685	Lv.6: 0.211/0.348
GM12878_insitu	Lv.2: 0.493/0.661	Lv.6: 0.125/0.222
HMEC	Lv.3: 0.523/0.687	Lv.6: 0.280/0.438
HUVEC	Lv.2: 0.587/0.740	Lv.5: 0.357/0.526
IMR90	Lv.1: 0.568/0.724	Lv.6: 0.129/0.229
K562	Lv.3: 0.645/0.784	Lv.6: 0.108/0.195
KBM7	Lv.3: 0.539/0.701	Lv.5: 0.220/0.360
NHEK	Lv.4: 0.582/0.736	Lv.6: 0.071/0.133

**Table 12:** Results of standard methods.

Method	MCF10A	MCF7	Change
Insulation Score	20	18	−10%
Directionality Index	201	172	−14%

**Table 13:** TAD hierarchy by radius.

Level	MCF10A	MCF7	Change
Lv.1	305	299	−2.0%
Lv.2	311	313	+0.6%
Lv.3	328	269	−18.0%
Lv.4	234	237	+1.3%
Lv.5	230	215	−6.5%
Lv.6	115	154	+33.9%

**Table 14:** Comparison with standard methods for isogenic pairs MCF-10A/MCF-7 (Jaccard/F1).

Level	MCF-10A (vs. Insulation)	MCF-10A (vs. DI)	MCF-7 (vs. Insulation)	MCF-7 (vs. DI)
Lv.1	0.038/0.073	0.417/0.588	0.027/0.052	0.406/0.577
Lv.2	0.022/0.043	0.445/0.616	0.018/0.035	0.446/0.617
Lv.3	0.017/0.033	0.436/0.607	0.026/0.052	0.441/0.612
Lv.4	0.019/0.038	0.338/0.506	0.025/0.048	0.290/0.450
Lv.5	0.058/0.110	0.336/0.503	0.049/0.094	0.245/0.393
Lv.6	0.102/0.184	0.152/0.264	0.066/0.123	0.190/0.319

**Table 15:** Comparing the best values.

Metric	MCF10A	MCF7
Best Jaccard vs. DI	0.445 (Lv.2)	0.446 (Lv.2)
Best F1 vs. DI	0.616 (Lv.2)	0.617 (Lv.2)
Best Jaccard vs. IS	0.102 (Lv.6)	0.066 (Lv.6)
Best F1 vs. IS	0.184 (Lv.6)	0.123 (Lv.6)

**Table 16:** Summary of key metrics for all 10 lines.

Cell Line	Status	IS TAD	BY TAD	Best Jaccard vs. DI	Best F1 vs. DI
4DNFIZGCA8AH	Healthy	7	78	0.521	0.685
GM12878	Healthy	8	74	0.493	0.661
HMEC	Healthy	8	87	0.523	0.687
HUVEC	Healthy	9	90	0.587	0.740
IMR90	Healthy	11	91	0.568	0.724
K562	Cancer	8	95	0.645	0.784
KBM7	Cancer	8	85	0.539	0.701
NHEK	Healthy	9	91	0.582	0.736
MCF-10A	Healthy	20	201	0.445	0.616
MCF-7	Cancer	18	172	0.446	0.617

**Table 17:** Healthy vs. cancer comparison (all 10 lines).

Metric	Healthy (Average)	Cancer (Medium)	Difference
IS TADs	10.29	11.33	−1.04
BY TADs	101.71	117.33	−15.62
Best Jaccard vs. DI	**0.553**	**0.543**	+0.010
Best F1 vs. DI	**0.710**	**0.700**	+0.010

**Table 18:** Comparison of results with the literature data for GSE63525 line.

This Study	References	Agreement
Jaccard vs. DI = 0.568	TADbit [9]: 0.45–0.55	Good
F1 vs. DI = 0.718	EmbedTAD [10]: 0.65–0.75	Good
Redistribution of hierarchy in cancer	[15]	Good
Degradation of the hierarchy	[12]	Good

## Data Availability

The analysis code is available at https://github.com/andytimoffilim/TAD_PNKR, accessed on 6 August 2026.

## Supplementary Materials

The following supporting information can be downloaded at: https://www.mdpi.com/article/10.3390/genes17080932/s1, Table S1: CTCF ChIP-seq enrichment at Poincaré TAD boundaries (K562, chr1, ±100 kb).
