# Supplementary material for: Applying the Poincaré Disc Method to Analyze the Hierarchical Structure of TADs in Hi-C Data
Source: Genes (Basel). 2026 Aug 10;17(8):932. doi: 10.3390/genes17080932 (PMC13512077; doi:10.3390/genes17080932)
Supplement: Supplementary file 1 [file genes-17-00932-s001.zip › genes-4456099-supplementary.pdf]

Supplementary Table S1. CTCF ChIP-seq enrichment at Poincaré TAD boundaries (K562, chr1, ±100 kb).

Metric	Small TADs (Lv.2–3)	Large TADs (Lv.5–6)	Random control
N boundaries	421	207	—
CTCF overlap (%)	88.1	72.5	78.1 / 79.2
Enrichment (binary)	**1.13× [1.06–1.20]**	0.91× [0.82–1.01]	—
Median distance (kb)	**16**	47	24
Distance enrichment	**1.48× closer**	0.52× (farther)	—
Fisher exact p	**7.4×10^{-5}**	0.96	—
Mann–Whitney U p	**8.0×10^{-4}**	0.997	—
Permutation p	**< 0.001**	1.0	—
